# A randomized controlled trial of directive and nondirective smoking cessation coaching through an employee quitline

**DOI:** 10.1186/s12889-016-3202-y

**Published:** 2016-07-11

**Authors:** Walton Sumner, Mark S. Walker, Gabrielle R. Highstein, Irene Fischer, Yan Yan, Amy McQueen, Edwin B. Fisher

**Affiliations:** Washington University School of Medicine, 660 S. Euclid Ave, Campus Box 8005, St. Louis, Missouri 63110 USA; Vector Oncology, Memphis, Tennessee USA; Winds of Change at Crosswinds, East Falmouth, Massachussetts USA; Peers for Progress; School of Public Health, University of North Carolina at Chapel Hill, Chapel Hill, North Carolina USA

**Keywords:** Employee health, Smoking cessation, Quitline, Directive coaching, Non-directive flexible coaching

## Abstract

**Background:**

Telephone quitlines can help employees quit smoking. Quitlines typically use directive coaching, but nondirective, flexible coaching is an alternative. Call-2-Quit used a worksite-sponsored quitline to compare directive and nondirective coaching modes, and evaluated employee race and income as potential moderators.

**Methods:**

An unblinded randomized controlled trial compared directive and nondirective telephone coaching by trained laypersons. Participants were smoking employees and spouses recruited through workplace smoking cessation campaigns in a hospital system and affiliated medical school. Coaches were four non-medical women trained to use both coaching modes. Participants were randomized by family to coaching mode. Participants received up to 7 calls from coaches who used computer assisted telephone interview software to track topics and time. Outcomes were reported smoking abstinence for 7 days at last contact, 6 or 12 months after coaching began. Both worksites implemented new tobacco control policies during the study.

**Results:**

Most participants responded to an insurance incentive introduced at the hospital. Call-2-Quit coached 518 participants: 22 % were African-American; 45 % had incomes below $30,000. Income, race, and intervention did not affect coaching completion rates.

Cessation rates were comparable with directive and nondirective coaching (26 % versus 30 % quit, NS). A full factorial logistic regression model identified above median income (odds ratio = 1.8, *p* = 0.02), especially among African Americans (*p* = 0.04), and recent quit attempts (*OR =* 1.6, *p* = 0.03) as predictors of cessation. Nondirective coaching was associated with high cessation rates among subgroups of smokers reporting income above the median, recent quit attempts, or use of alternative therapies. Waiting up to 4 weeks to start coaching did not affect cessation. Of 41 highly addicted or depressed smokers who had never quit more than 30 days, none quit.

**Conclusion:**

Nondirective coaching improved cessation rates for selected smoking employees, but less expensive directive coaching helped most smokers equally well, regardless of enrollment incentives and delays in receiving coaching. Some subgroups had very low cessation rates with either mode of quitline support.

**Trial registration:**

ClinicalTrials.gov NCT02730260, Registered March 31, 2016

## Background

High smoking prevalence in low-income populations contributes to economic and ethnic disparities in health outcomes. Worksite smoking cessation programs can reach low-income working populations.

Telephone quitlines increase smoking cessation rates relative to no intervention, with estimated long-term abstinence rates of 13 % [1, 2]. Evidence supports a dose-response effect of multiple call-backs, with little difference among counseling strategies or materials [3, 4]. Other recommended elements include: increased total contact, providing practical counseling (problem solving/skills training), providing social support as part of treatment, supporting pharmacotherapy, and assistance in securing social support outside of treatment [1]. Quitlines callers usually are in the transtheoretical model’s contemplation or preparation stage of change [5].

Social support modes may be directive or nondirective [6]. Directive support is prescriptive: The provider tells the recipient what to do, or even what to feel (e.g., “Look on the bright side”). In nondirective support the provider listens, leaves responsibility for tasks and decisions with the recipient, and assists when asked. Nondirective support entails eliciting and accepting recipients’ feelings (e.g., “That must be upsetting”). In contrast to motivational interviewing, nondirective support is more cooperative and accepting of feelings and choices, and less intent on influencing the recipient to pursue a goal [7, 8]. Nondirective family support has been reported to improve morale, while directive support can be isolating [9]. Nondirective support has been associated with better outcomes in some settings [10–12], suggesting that “health outcomes might depend upon the type rather than the amount of support provided to recipients [12].” However, other studies favor directive support [13].

The value of social support modes could be contextual. Recipients’ mode of interaction with advisors and authorities, experience with behavior changes, and internal motivation could affect reactions to support modes. Nondirective coaching of parents improved asthmatic children’s outcomes in disadvantaged families [8], and generated interest in testing this coaching mode in a quitline recruiting from the working poor.

Directive interventions are easily standardized and replicated on a large scale. Nondirective support may require more highly skilled coaches to handle diverse topics that recipients might broach, and therefore could be more expensive. Thus comparisons of nondirective and directive coaching are of interest for health promotion interventions that use social support, including quitlines.

We studied nondirective versus directive telephone quitline coaching offered through worksites, to test the hypothesis that nondirective coaching mode improves smoking cessation rates among ethnic minorities and the working poor.

## Methods

### Research setting

Call-2-Quit was a prospective randomized controlled trial comparing directive and nondirective quitline coaching, offered at two institutions headquartered in St. Louis, Missouri. The first was the BJC Health Care System (the hospital system), comprising 13 campuses in Missouri and Illinois, with 25,000 employees, of whom an estimated 25 % (6250) smoked. Employees and spouses could enroll in Call-2-Quit from November 2005 to March 2008 (funding ended 6 months later).

To increase enrollment, the program was offered to the 8850 employees and spouses of the Washington University School of Medicine (the school) from November 2006 to March 2008. Due to strikingly low enrollment, a supervisor survey was conducted at the school to estimate smoking prevalence among employees.^1^

### Recruitment

Participants called a toll free number (866-902-QUIT) to initiate enrollment. An automated 300-word announcement provided informed consent and 24-h access. Smokers in the contemplation, preparation, or action stage of change could begin coaching if they agreed to be randomized to a coaching mode.

Both organizations promoted Call-2-Quit through multiple channels including health fairs, employee web sites, employee news, promotional posters, fliers, and department managers. Each organization promoted Call-2-Quit to help smokers adapt to tobacco control policies implemented during the trial.

In 2006, the hospital system implemented health insurance discounts of $10/month for employees who committed, during open enrollment in November, to pursue several health promoting activities. Smokers obtained the discount by “enrolling” in a qualifying smoking cessation program, such as Call-2-Quit, before the following April. Smokers who completed a screening telephone interview obtained the discount: they did not have to start coaching or stop smoking. The health insurance discount increased to $15/month for 2007 and 2008. Health insurance covered FDA-approved smoking cessation aids, usually with a copayment.

The school implemented a tobacco-free campus policy on April 2^nd^, 2007. The school offered discounted nicotine replacement products ($15 for a 6 week supply) to employees enrolled in smoking cessation programs including Call-2-Quit. However, the hospital maintained designated smoking areas near the school during the study.

### Enrollment, randomization, and withdrawal

Four part-time coaches assisted up to 20 participants each at any given time. The program could enroll 7-8 smokers each week, and used a waiting list as needed.

The unit of randomization was the family. After baseline data were entered, members of a previously randomized family were assigned to the family coaching mode. New families were randomized to directive or nondirective coaching mode in a 1:1 ratio, based on a randomization table, when the first member enrolled. Consent to randomization was required to participate. New enrollees were assigned to the least busy coach, who conducted all coaching calls. Each coach worked in both nondirective and directive coaching modes. Coaches attempted to schedule regular telephone meetings with participants. Participants were excluded from further coaching at their request, after 10 consecutive or 15 unsuccessful attempts to arrange calls, 120 days after the first call, or after the 7^th^ call.

### Interventions

Coaches were nonmedical adult women trained to use Computer Assisted Telephone Interview (CATI) software to guide and document both directive and nondirective coaching interactions. GH instructed each coach in both coaching modes.^2^ Each coach then managed 5-6 participants as training cases under close supervision by GH before coaching the participants reported here. Coaches were taught to table unfamiliar clinical and counselling questions which arose during nondirective coaching, then discussed these with MW or WS at bi-weekly team meetings, then offered advice to participants during subsequent calls.

Directive and nondirective coaching shared these features:

- Seven calls over 56 to 90 days
- Coach reviews status of previous weeks’ goals.
- Coach encourages coverage of six key steps in smoking cessation
  1. Set a specific quit date
  2. Discuss nicotine replacement and other drug therapy
  3. Consider other smoking cessation resources, such as a smoking cessation group
  4. Break up smoking patterns [14]
  5. Identify and develop plans for coping with circumstances likely to cause relapse
  6. Seek cooperation and encouragement from friends and family
- Coach documents goals for the ensuing week and schedules next call.

Nondirective coaching included these distinctive features:

- Seven calls planned over 90 days, as convenient to smoker and coach
- Quit date set according to individual preference
- Coach offers topics at each call, smoker selects one, or may choose a novel topic

Directive coaching included the following distinctive features:

- Calls scheduled about one week apart, except calls #4 and #7
- Fixed topic schedule:

- Call #1: Describe directive calls; assess smoking history, quitting history; recall past success to enhance self-efficacy; encourage self-image as a nonsmoker; introduce motivation and barriers worksheet
- Call #2: Assess weekly progress; review motivation and barriers worksheet; strategize on dealing with barriers; discuss nicotine replacement therapy and bupropion; introduce tracking smoking worksheet; discuss breaking links in smoking patterns.
- Call #3: Review tracking smoking; discuss breaking links in smoking patterns, eliminating cues to smoke, diet and weight gain, and social support; review motivation and self-image; set and prepare for quit day within 2 weeks.
- Calls #4-7: Call #4 closely follows quit date. Assess status (did not try to quit, tried and relapsed, or succeeded); get quit day synopsis; praise efforts; review motivation, barriers, strategies, withdrawal symptoms, and social support; invite request for more information.
  Set a new quit date if smoking.
  Set a reward and encourage if not smoking.
- Call #7: Scheduled 2 weeks after call #6. In addition to the above, remind that this is the final call; offer online resources for future use.

- Individuals started with call #4 if they quit smoking before beginning coaching.

### Intervention integrity

Coaches recorded all calls and a subset were reviewed with GH. Coaches received weekly feedback regarding implementation of directive and nondirective coaching modes.

The CATI database program tracked telephone conversations. For directive coaching, the program displayed topical screens for that call. For nondirective coaching, the program listed all defined topics, allowing the coach to select relevant screens for topics chosen by the participant. The database recorded time spent viewing each topic screen.

Social support received from coaches was evaluated using the social support inventory [13], which generates scores for both nondirective and directive support. Social support from other sources also was evaluated at baseline and follow-up.

The planned analysis of social support inventory by coaching mode showed no difference, prompting an analysis of coaching language recorded in transcripts. Some series of calls were transcribed during the trial (12 directive series, 15 nondirective series, 190 distinct calls). A software scan [15] was done between April and October 2014, using regular expressions to detect coaching statements suggesting the mode. Statements that offer control to the participant, such as “what would you like to talk about?” suggest nondirective mode. Statements that redirect conversation, such as “let’s talk about…” suggest directive mode. We also screened coach and participant comments for:

- Instructions (you should|must|need to|ought to|have to…)
- Questions of fact (who|how|what|why|where|when…?)
- Suggestions posed as questions (could you…?)
- Suggestions (you could…)
- Responses to questions (a response after any kind of question)
- Active listening (mmmhmm|okay|right…)
- Celebration (congratulations|good work|…)
- Empathy (oh dear|that’s too bad|…) (“I’m sorry” usually indicated confusion, not empathy.)

### Data collection

A research team member other than the participant’s coach administered baseline, 6-month, and 12-month surveys. The baseline survey included height and weight, the Fagerström test for nicotine dependence (FTND) [16]; the sharing subscale of the patient practitioner orientation scale (PPOS) [17], a measure of interest in patient-centric care; the relapse situation efficacy questionnaire (RSEQ) [18]; appetitive and aversive scores from the brief questionnaire of smoking urges (QSU) [19]; Eight items of the Patient Health Questionnaire (PHQ) depression severity measure (omitting suicidal ideation) [20]; spouse and family member smoking history, and personal smoking history. The CATI program captured process variables including call number, call duration, and time spent on each topic discussed, and allowed coaches to record goals and achievements at each call. Follow-up surveys collected outcome data and additional process variables including timing and degree of lapses and relapses, and use of nicotine replacement therapy, bupropion, varenicline, and alternative treatments.

### Measures of smoking cessation

Self-reported smoking was assessed at baseline and 6 and 12 months later. Smoking cessation was defined as answering “no” to the question, “Have you smoked any in the last 7 days?” at last contact. Individuals who were not reached at either 6 or 12 months were counted as smokers. We attempted to obtain saliva cotinine assays by mail from participants who reported quitting. In the final year of data collection, we attempted instead to collect a witnessed cheek swab.

### Analysis

Power estimates assumed recruitment of 15 % of smokers at the hospital (850-900 participants), directive cessation rates of 13 % based on quitline data [1, 2], and nondirective cessation rates of 19-25 %, yielding power of 0.65 to 0.99.

Total contact time, call scheduling, and use of any smoking cessation aids were compared across coaching modes using t-tests and chi-square tests (JMP 11, SAS Institute), to demonstrate comparability. Call content (topics covered), language, and perceived social support were compared to evaluate distinctiveness of the interventions.

The primary analyses used logistic regression models to examine coaching completion, follow-up data collection, and the a priori primary outcome of smoking cessation rates by coaching mode, race, and income. Coaching was classified as completed (all seven calls) or not. Follow up data collection (retention) was classified as occurring at either 6 or 12 months, or not. Income was classified as low income (at or below the median value of $30,000) or higher income (>$30,000). Missing income data was managed with listwise deletion in the primary analysis. In a confirmatory analysis, single imputation of missing income data in 29 records was done using CART models.^3^ Race analysis was restricted to self-identified European-Americans and African-Americans. One variable, quit attempts in the year preceding coaching, was not balanced by randomization, and was added to the model as another binary variable (occurring or not). A full factorial model was generated, then pruned by removing interaction terms with parameter estimate p-values >0.10. Secondary analyses explored dose-responsiveness (number of calls completed), timing of lapses, delays in coaching, and concurrent pharmacotherapy as process variables that might influence smoking cessation rates.

Exploratory analyses were performed to identify subgroups at high and low risk to continue smoking. First, we evaluated subgroups suggested by the primary analysis. Second, we used classification and regression trees (CART) to screen for subgroups, defined by up to 4 baseline or baseline and process variables excluding coaching mode, with high or low cessation rates. The effectiveness of coaching modes within subgroups was evaluated using chi-square tests.

## Results

### Enrollment, randomization, and completion

**Fig. 1:**
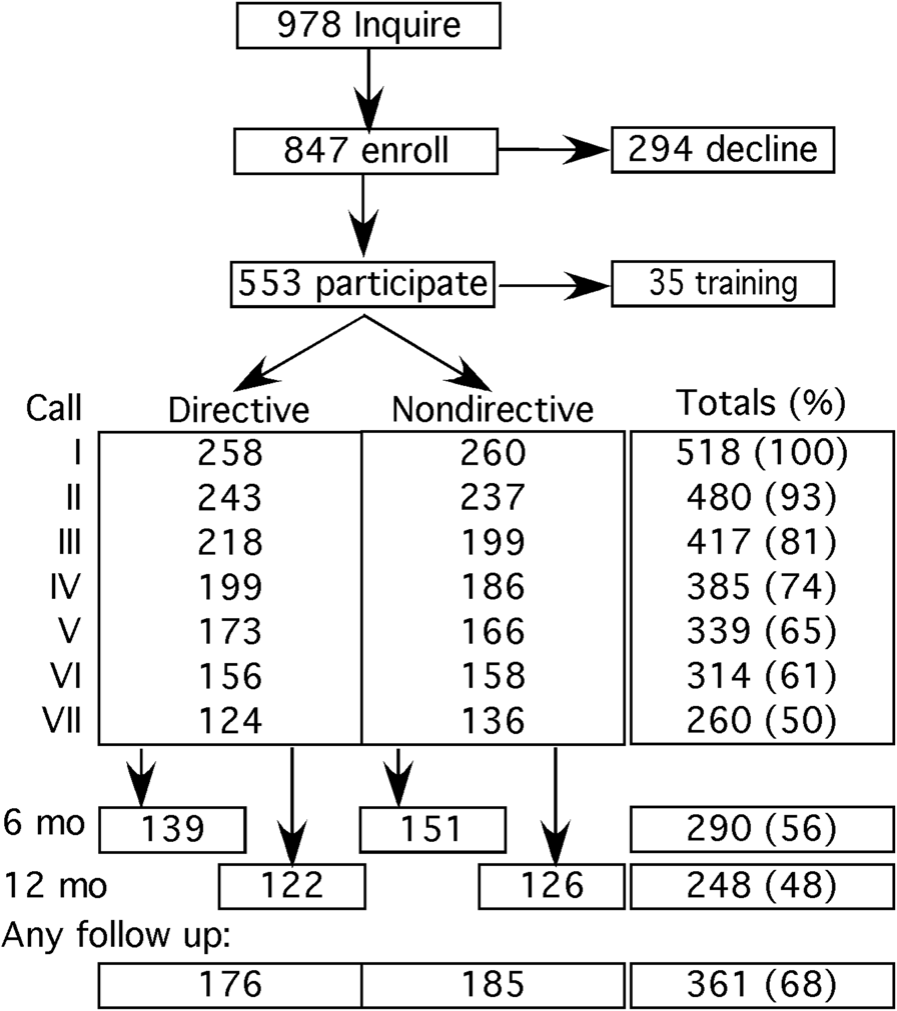
Consort diagram

The program received 978 inquiries over 29 months and enrolled 847 into the program; of these, 553 completed at least one coaching call (Fig. 1). Enrollees who recorded baseline data but did not participate in coaching were not significantly different from participants in age, race, income, sex, marital status, current smoking, age when smoking began, cigarettes smoked per day, or FTND.

**Fig. 2:**
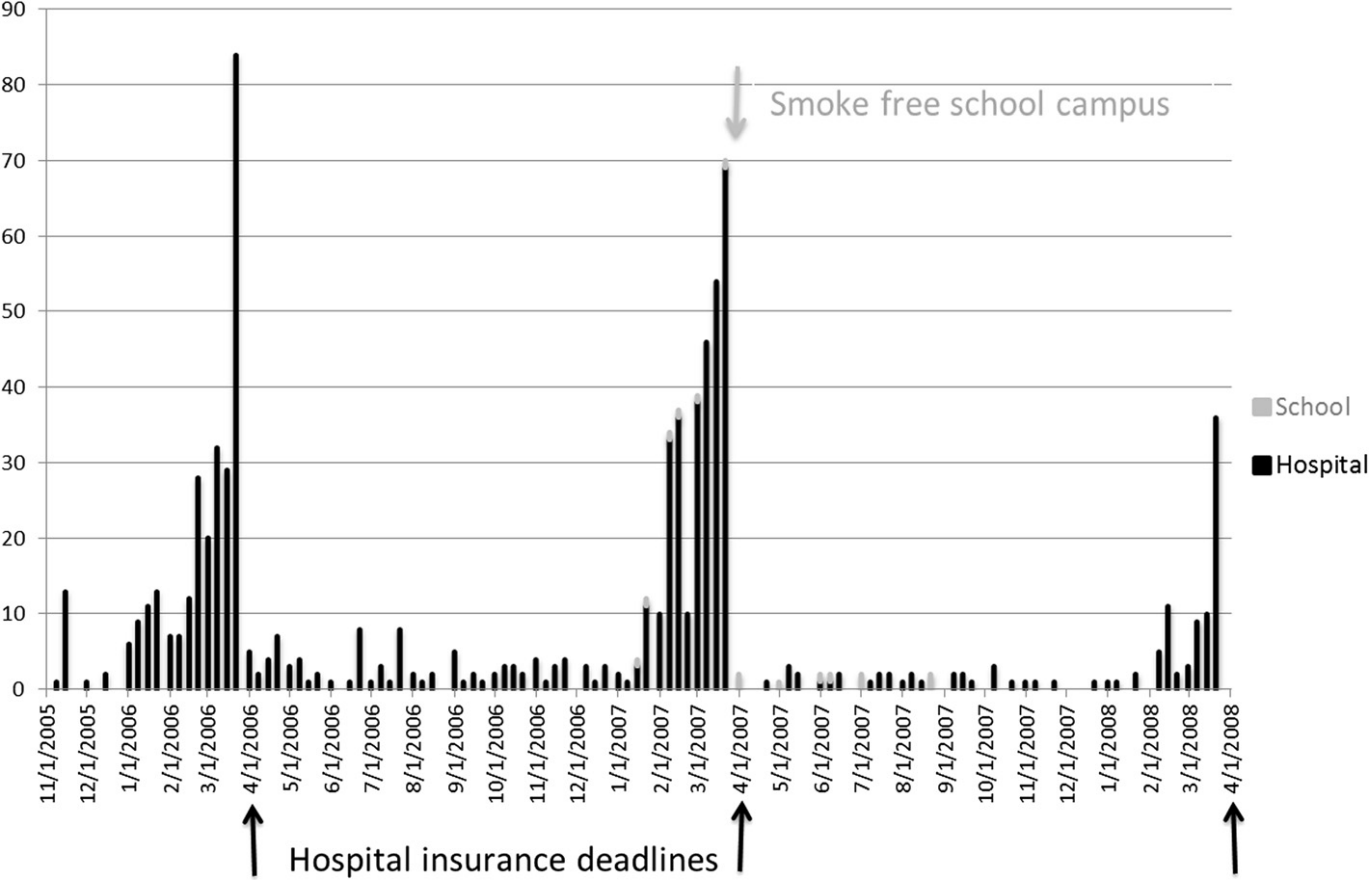
Weekly enrollment data from two work sites with different tobacco policies. The main work site data and events are in black: annual deadlines for obtaining an insurance discount by enrolling in a smoking cessation program like Call-2-Quit generated enrollment spikes in the first two years of the quitline. The secondary work site, in gray, became smoke free on April 2, 2007, without changing interest in the program. Over the course of the program, a similar, small fraction of the smokers at each of the two work sites enrolled each year

The hospital system’s health insurance incentive caused strikingly seasonal recruitment (Fig. 2). Twenty smokers enrolled from the medical school. The smoke-free campus deadline did not affect enrollment. However, the supervisor survey implied that only about 530 medical school employees smoked.^1^ Consequently, estimated enrollment rates were similar at the medical school (20 enrollees/530 eligible smokers/17 months = 0.22 % of eligible smokers per month) and the hospital (533/6250/29 = 0.29 % per month).

Nondirective (*N* = 260) and directive (*N* = 258) groups were similar on most attributes (Table 1). Two-thirds were female, less than one-quarter were African-American, and 45 % reported personal income below $30,000. However, participants randomized to nondirective coaching reported more quit attempts in the previous year.

**Table 1:** Participant characteristics

	Directive	Flexible	*p*
Demographics			
N	258	260	
Age	47 (38, 55)	47 (39, 53)	0.8
% European-Americans	78	78	0.99
% Female	66	66	0.9
Clinical status			
Patient Practitioner Orientation Scale	3.3 (3.0, 3.7)	3.3 (3.0, 3.7)	0.98
Patient Health Questionnaire (8 items)	5 (3, 8)	5 (2, 8)	0.8
Body Mass Index	28 (24, 32)	27 (24, 31)	0.5
Social situation			
% Single	17	18	0.9
% Married	55	56	
% Divorced	21	19	
% With smoking spouse	31	38	0.4
% Employed full time	65	73	0.8
% Retired	15	10	
% High school or less	30	29	0.9
% Finished college	27	29	
% Low job rank (e.g. custodial)	35	27	0.2
% High job rank (e.g. managerial)	36	40	
% With personal income <30 K	46	39	0.3
% With personal income >60 K	18	19	
% With family income <30 K	15	17	0.3
% With family income >60 K	52	57	
% Christian religious affiliation	78	75	0.5
Smoking history			
Age of smoking initiation	15 (13,17)	15 (13,17)	0.8
Age when first smoking regularly	18 (16, 20)	18 (16, 20)	0.4
% Smoking at first call	53	58	0.4
# Cigarettes per day	11 (9, 20)	10 (7, 20)	0.4
Fagerström score	3 (1, 5)	3 (1, 4)	0.9
% Attempting to quit in past year	61	67	0.1
# Quit attempts in past year	1 (0, 2)	**1 (0, 3)**	**0.05**
Relapse Situation Efficacy Questionnaire	2.4 (2.1, 2.7)	2.4 (2.1, 2.6)	0.5
Most days without smoking in past year	61 (7, 274)	31 (7, 365)	0.8
Social Support Directive Mean	3.6 (3.0, 4.2)	3.5 (3.0, 4.1)	0.07
Social Support Flexible Mean	4.4 (4.0, 4.8)	4.2 (3.9, 4.8)	0.1
QSU – Appetitive	2.5 (2.0, 3.2)	2.3 (1.7, 3.2)	0.4
QSU – Aversive	1.7 (1.3, 2.5)	1.7 (1.3, 2.5)	0.96

Continuous values are given as: median (25^th^ percentile, 75^th^ percentile). Bold data are the higher value when the coaching modes are significantly different at *p* < 0.05. Bold *p* values are less than or equal to 0.05

Completion rates did not differ by coaching mode, income, or race. Over half completed all coaching calls. Attrition between calls averaged 5 to 10 %, except for an unexplained loss of nearly 20 % between calls 2 and 3 during nondirective coaching. Six and/or 12-month follow up data were obtained from 64 % of European American and 76 % of African American participants (*p* = 0.02), with no difference between coaching modes.

### Intervention integrity

Total contact time averaged 98 min, with no difference between coaching modes (Table 2). Interventions also were similar in call timing, except that the interval between the 6^th^ and 7^th^ calls was longer in the nondirective coaching mode.

**Table 2:** Comparison of coaching mode characteristics

	Directive	Nondirective	*p*
Call timing (days after baseline call)			
Call I	12 (7, 19)	12 (7, 20)	0.8
Call II	22 (15, 34)	22 (15, 37)	0.5
Call III	36 (27, 50)	37 (27, 55)	0.5
Call IV	51 (39, 71)	52 (40, 71)	0.8
Call V	63 (49, 81)	65 (50, 84)	0.3
Call VI	75 (57, 96)	78 (63, 101)	0.06
Call VII	88 (71, 105)	**94 (74, 119)**	**0.04**
Call length (minutes)			
Call I	15 (11, 21)	**19 (13, 26)**	**0.0003**
Call II	**23 (17, 30)**	18 (12, 26)	**0.0001**
Call III	**22 (15, 30)**	18 (12, 25)	**0.002**
Call IV	14 (9, 19)	**17 (10, 25)**	**0.018**
Call V	13 (8, 18)	14 (8, 34)	0.2
Call VI	14 (8, 28)	17 (9, 39)	0.2
Call VII	12 (7, 18)	12 (7, 18)	0.9
Total contact time	99 (58, 138)	97 (53, 140)	0.6
Calls completed			
# of calls completed	7 (4, 7)	7 (3, 7)	0.2
Total time spent on topic (minutes)			
Future contacts	0.8 (0.6, 1)	**1.2 (0.9, 1.5)**	**<0.0001**
Smoking history	**1.5 (1, 2)**	1.4 (0.8, 2.2)	**0.03**
Quitting history	**1.4 (0.9, 2.2)**	1 (0, 1.9)	**<0.0001**
Past success	**1.6 (0.9, 2.4)**	0 (0, 0.9)	**<0.0001**
Self image	**1.5 (0.9, 2.5)**	Max 19.3	**<0.0001**
Motivator/barrier worksheet	**2.3 (1.5, 4)**	Max 0	**<0.0001**
Self efficacy	**0.6 (0.2, 1.3)**	0 (0, 1.5)	**<0.0001**
Motivation	1.8 (1, 3.3)	**3 (0.8, 8)**	**0.0001**
Barriers	2.3 (0.8, 4.4)	1.6 (0, 4.9)	0.1
Dealing with barriers	1.7 (0.5, 3)	1.2 (0, 5.7)	0.3
Pharmacotherapy	6 (2.1, 9)	**6.9 (0.8, 14)**	**0.02**
Track smoking	**2.3 (1.2, 3.8)**	0 (0, 1.8)	**<0.0001**
Review tracking smoking	**1.2 (0.4, 2.5)**	0 (0, 0.09)	**<0.0001**
Break smoking links	**0.7 (0.05, 1.8)**	0 (0, 1.8)	**<0.0001**
Breaking links smoking patterns	**2.4 (0.3, 5.9)**	0 (0, 3.4)	**<0.0001**
Eliminating cues to smoke	**2.3 (0.06, 4.2)**	0 (0, 1.7)	**<0.0001**
Diet and weight gain	1.3 (0.02, 2.7)	0.6 (0, 4.5)	0.2
Social support	**4.4 (1.4, 7.8)**	1.7 (0, 6)	**<0.0001**
Review motivations quitting	**4.6 (1.6, 8.8)**	0 (0, 0.9)	**<0.0001**
Set new quit date	**2 (0.2, 5.4)**	0 (0, 0.7)	**<0.0001**
Preparation for quit day	**0.3 (0.01, 1.3)**	0 (0, 0) Max 9	**<0.0001**
Introduction to calls 4-6, 7	**0.6 (0, 1.2)**	0 (0, 0) Max 0	**<0.0001**
Describe quit day	**1.3 (0, 2.9)**	0 (0, 0) Max 10	**<0.0001**
Praise participant efforts	**0.6 (0, 1.5)**	0 (0, 0) Max 28	**<0.0001**
Review barrier strategies	**3.8 (0, 8.5)**	0 (0, 1.7)	**<0.0001**
Need more information	**0 (0, 1.3)**	0 (0, 0) Max 12	**<0.0001**
Setting rewards	**0 (0, 2.8)**	0 (0, 1.2)	**0.0008**
Withdrawal symptoms	0 (0, 2.4)	0 (0, 1.9)	0.2
Be careful	**0 (0, 2.5)**	0 (0, 0) Max 32	**<0.0001**
Final reminder	**0.03 (0, 0.3)**	0 (0, 0.4)	0.06
Other resources for quitting	**0 (0, 1.4)**	0 (0, 0) Max 10	**<0.0001**
Final good-bye	**0 (0, 1.2)**	0 (0, 0) Max 0	**<0.0001**
Responding to life events	Max 0	**0 (0, 0) Max 48**	**<0.0001**
Caller’s coach social support inventories (CSSI)			
CSSI - Directive	3.6 (2.9, 4.2)	3.6 (2.8, 4.1)	0.6
CSSI - Nondirective	4.6 (4, 5)	4.6 (3.9, 4.9)	0.4
Types of statements coaches made (mean number per call)			
Redirection	0.03	0	0.06
Offer control	0.2	**0.5**	**0.0001**
Factual questions	**15**	13	**0.04**
Suggestive questions	**1.2**	0.8	**0.05**
Suggestions	**6.4**	5.1	**0.04**

Continuous values are given as: median (25^th^ percentile, 75^th^ percentile). Max X indicates that the maximum reported value is X when the 75^th^ percentile value is 0. Bold data are the higher value when the coaching modes are significantly different at *p* < 0.05. Bold p values are less than or equal to 0.05

The interventions differed significantly in time spent on 28 of the 33 discussion topics, and in four kinds of statements made by coaches (Table 2). The nondirective group spent more time discussing pharmacotherapy, motivation, and life events: a few reported significant social and financial stress, including immediate external health threats. The groups spent equal time discussing barriers, diet, and withdrawal. The directive group spent more time on all other topics. Nevertheless, participants rated the nondirective and directive dimensions of social support received from coaches as equal in the two coaching modes, prompting the transcript analysis. In directive mode, coaches asked more factual questions and offered more advice. In nondirective mode, coaches offered control to callers more often. Coaches rarely redirected conversations, but only did so in directive mode.

Numbers of cessation attempts (56 % of participants quit for 23 h or more), lapses during (42 % of attempts) or after (92 % of attempts) coaching, and use of pharmacologic treatments (58 % of participants) were similar across coaching modes. Some participants attributed odd pharmacologic advice to their physicians: coaches offered corrective conventional advice in both modes. Coaches supported varenicline use in both modes after it was approved in 2007.

### Smoking cessation

Self-reported smoking cessation at last contact provided our most complete outcome measure. Saliva cotinine kits were almost never returned. Most participants declined cheek swab sampling at worksites, often explaining that meeting would interfere with work.

Self-reported smoking cessation was comparable with directive and nondirective coaching for all enrolled participants at 6 months (25 % vs 23 %, chi square *p* = 0.7), 12 months (19 % vs 22 %, *p* = 0.5), and last contact (26 % vs 30 %, *p* = 0.4). The logistic regression model did not identify coaching mode or any of its interaction terms as predictors of smoking cessation (Table 3). It did identify income above the median (Odds Ratio 1.8 [95 % confidence interval 1.08, 3.2], *p* = 0.02), prior cessation attempts (OR 1.6 [1.06, 2.6], *p* = 0.04), and an interaction between race and income (*p* = 0.03) as predictors of cessation. Among African Americans, above-median income predicted cessation more strongly (OR 3.2 [1.2, 8.0] *p* = 0.02).

**Table 3:** Participants not smoking at last contact (%), by coaching mode

	Directive	ND	Trends and significant effects
All participants	26	30	None
Logistic regression model with Mode, Race, Income, Prior Quit, and interactions	26	30	Income (*OR =* 1.8);
			Prior Quit (*OR =* 1.6);
			Income x Race
Subgroups related to primary hypothesis			
European-American (EA)	26	30	None
African-American (AA)	30	29	
Personal income <30 K	27	23	Above median income + ND > remainder (chi square, *p* = 0.03)
Personal income >30 K	25	35	
No prior quit attempts	23	20	Prior quit + ND > remainder (chi square, *p* = 0.03)
At least one quit attempt	27	34	
Income <30 K and AA race	28	17	Above median income > below (chi square, *p* = 0.014)
Income >30 K and AA race	40	52	
No prior quit or low income	25	24	Prior quit + above median income + ND > remainder (chi square, *p* = 0.004)
Prior quit and high income	26	40	
Subgroups by delay in coaching			
1 week wait for coaching	30	24	None
2 week wait for coaching	20	31	
3 week wait for coaching	28	41	
4 + week wait for coaching	29	27	
Worksite subgroups (incentives)			
Hospital (insurance discount)	29	31	None
School (smoke-free campus)	25	33	
Subgroups using smoking cessation treatments, among those with follow-up data			
No treatment	43	40	None
Nicotine replacement	37	47	None
Bupropion	40	43	None
Varenicline	35	48	None
Auricular therapy	21	38	None
Hypnosis	8	37	ND > Directive? (*p* = 0.06)

*AA* African-American, *d* days, *EA* European-American, *FTND* Fagerström Test for Nicotine Dependence, *K* thousands, *ND* Nondirective, *OR* Odds Ratio, *PHQ* 8-items of the Patient Health Questionnaire, *RSEQ* Relapse Situation Efficacy Questionnaire

Nevertheless, the nondirective coaching mode appeared to benefit two subgroups. First, above median income participants receiving nondirective coaching quit more often (35 %) than the remainder (24 %, chi-square *p* = 0.03). Second, participants who had attempted to quit in the prior year and received nondirective coaching quit more often (34 %) than the remainder (23 %, chi-square *p* = 0.03).

### Secondary results

Coaching queues did not interfere with cessation rates, but extended participants’ opportunity to quit before enrollment. Most participants promised in November that they would begin a smoking cessation program within 4 months, and enrolled the following February or March. Those enrolling in March often waited an additional 1 to 4 weeks to begin coaching, due to staffing limitations. Waiting had no effect on cessation rates (Table 3). However, at baseline 33 participants had quit smoking and another 25 smoked only 1 to 3 cigarettes daily. Their FTND scores reflect smoking behavior at baseline, not in November. Of these 58 (11 % of the 528 participants), 31 (53 %) had quit at last contact, accounting for 21 % of successful cessation attempts. These participants were evenly distributed between modes. Repeating the primary logistic regression analysis with only current smokers at baseline yielded odds ratios of 1.6 for income above the median (*p* = 0.09) and 1.5 for prior cessation attempts (*p* = 0.07), and among African Americans, an odds ratio of 2.6 for above median income (*p* = 0.06).

Pharmacologic interventions and alternative therapies were oddly unhelpful with directive coaching (Table 3). Among the subset of participants with follow-up data, the smoking cessation odds ratios associated with receiving conventional pharmaceutical interventions were 1.4 (95 % CI 0.8 to 2.6) with nondirective coaching, consistent with previous reports of 1.4 to 1.9 [21], but only 0.7 (0.4 to 1.36) with directive coaching (chi-square, *p* = 0.01). Time spent discussing pharmacotherapy did not predict cessation.

### Exploratory results

CART analyses identified subgroups likely to either continue smoking or quit (Tables 4 and 5). Highly addicted or depressed participants who had relapsed in less than 30 days during past cessation attempts seldom succeeded in quiting. Among participants who had not previously quit for at least 30 days, only 17 % quit (Table 4). Among these, none of those with an FTND above 6 reported quitting; among those with lower FTND scores, participants who were younger and depressed or older with low wages were unlikely to quit. Participants who did not complete coaching and had quickly relapsed in the past were similarly unsuccessful, especially if depressed (Table 5).

**Table 4:** Classification and Regression Tree predicting smoking cessation success rates from baseline data

Longest quit < 30 d (*N* = 222) 17 % quit at last contact	FTND ≥ 7 (24) 0 %		
	FTND < 7 (198) 19 %	Enroll age < 54.6 y (153) 14 %	PHQ ≥ 6 (71) 7 %
			PHQ < 6 (82) 21 %
		Enroll age ≥ 54.6 y (45) 36 %	Income < $45 k (16) 6 %
			Income > $45 k (29) 52 %
Longest quit ≥30 d (296) 36 %	RSEQ < 3.3 (272) 33 %	PPOS ≥ 4.2 (9) 0 %	
		PPOS < 4.2 (263) 34 %	Enroll age < 26 (8) 0 %
			Enroll age ≥ 26 (255) 35 %
	RSEQ ≥ 3.3 (24) 75 %	BMI ≥ 25 (16) 62 %	Divorced (6) 17 %
			Single/Married (10) 90 %
		BMI < 25 (8) 100 %	

Among all participants (*N* = 518), 28 % quit at last contact. Each cell is a subset of the cell to its left, split on the indicated variable and value, and shows the number of persons classified (N), and the percentage who had quit at last contact (%)

*BMI* Body Mass Index, *FTND* Fagerström Test for Nicotine Dependence, *PHQ* 8-items of the Patient Health Questionnaire, *PPOS* the sharing subscale of the Patient Practitioner Orientation Scale, a measure of interest in shared decision making, *QSU.AV* Aversive subscale of the Questionnaire of Smoking Urges, *RSEQ* Relapse Situation Efficacy Questionnaire

**Table 5:** Classification and regression tree predicting smoking cessation success rates from baseline and process data

Did not complete all calls (*N* = 238) 14 % quit at last contact	Longest quit < 61 d (124) 4 %	PHQ ≥ 4 (79) 0 %	
		PHQ < 4 (45) 11 %	Key steps < 4 (34) 0 %
			Key steps ≥ 4 (11) 45 %
	Longest quit ≥ 61 d (114) 25 %	RSEQ < 2.7 (83) 16 %	Start age ≥ 17 (24) 0 %
			Start age < 17 (59) 22 %
		RSEQ ≥ 2.7 (31) 48 %	Calls ≥ 33 min (23) 35 %
			Calls < 33 min (8) 87 %
Completed all calls (280) 40 %	PPOS ≥ 3.8 (51) 18 %	RSEQ < 2.1 (13) 0 %	
		RSEQ ≥ 2.1 (38) 24 %	QSU.AV < 1.5 (16) 6 %
			QSU.AV ≥ 1.5 (22) 36 %
	PPOS < 3.8 (229) 45 %	FTND ≥ 6 (30) 17 %	QSU.AV < 3.8 (25) 8 %
			QSU.AV ≥ 3.8 (5) 60 %
		FTND < 6 (199) 49 %	RSEQ < 3.3 (185) 46 %
			RSEQ ≥ 3.3 (14) 93 %

Among all participants (*N* = 518), 28 % quit at last contact. Each cell is a subset of the cell to its left, split on the indicated variable and value, and shows the number of persons classified (N), and the percentage who had quit at last contact (%)

*BMI* Body Mass Index, *FTND* Fagerström Test for Nicotine Dependence, *PHQ* 8-items of the Patient Health Questionnaire, *PPOS* the sharing subscale of the Patient Practitioner Orientation Scale, a measure of interest in shared decision making, *QSU.AV* Aversive subscale of the Questionnaire of Smoking Urges, *RSEQ* Relapse Situation Efficacy Questionnaire. Six key steps are described in the Interventions section of the Methods. Start age is the age when the participant first smoked

In contrast, high self-efficacy and a history of longer abstinence predicted high cessation rates. Among those who had previously quit for 30 days or more, high self-efficacy was associated with reported quitting. Another successful group comprised participants who completed calls and had low interest in patient centered care: Cessation rates were 45 % in the subgroup of participants who completed all 7 calls and had a low interest in shared decision making (PPOS <3.8).

In these subgroups, cessation rates are significantly different from the remainder of the group (Chi-square, *p* < 0.01), and are not significantly affected by coaching mode.

## Discussion

This randomized controlled trial compared directive and nondirective smoking cessation coaching modes, delivered through an employer-endorsed telephone quitline. Most participants were hospital system employees motivated by a health insurance discount. African-American employees enrolled in numbers reflecting local demographics. We observed comparable rates of program completion and smoking cessation among European Americans, African Americans, and employees with annual incomes above and below the median of $30,000. Participants reported relatively high rates of smoking cessation, possibly reflecting persuasive insurance incentives and relatively high use of pharmacotherapy (>30 %).

We found no main effect of coaching mode and no interactions between coaching mode and income or race in the primary logistic regression analysis. Nevertheless, nondirective coaching mode may improve cessation rates in subgroups including those with above median income, recent cessation attempts, and issues that directive scripts did not anticipate, such as new or alternative smoking cessation therapies. If so, adding specific topics and content to the directive coaching scripts might improve outcomes. Directive coaching did not benefit any identified subgroup, but performed well for most smokers. Compared to nondirective coaching, directive coaching may be less expensive to provide if less skilled coaches are employed.

Insurance incentives created seasonal enrollment surges that overwhelmed quitline resources, but queuing employees for up to 4 weeks did not reduce cessation rates. Surges in quitline calls can occur with rising cigarette taxes [22] and smoking bans [23]. Quitlines that implement queues in these situations may achieve their usual smoking cessation rates despite delays in coaching.

Delays between commitments and coaching allowed participants to taper or quit before coaching, and 1 out of 9 did so. These participants accounted for 1 out of 5 successes. Quitlines that provide directive coaching to queued clients should design alternative call schedules to support individuals who recently quit.

Two subgroup observations were noteworthy. First, some circumstances presage great difficulty with smoking cessation. In this study, a history of short quit attempts and either high nicotine dependence or depressive symptoms was associated with very low quit rates. These differences may reflect nicotinic acetylcholinergic receptor [24] or dopamine receptor [25] genotypes that are resistant to smoking cessation. The increasing prevalence among smokers of such genotypes [26] eventually should limit the effectiveness of conventional smoking cessation programs [27]. For smokers with these genotypes, harm reduction may be an effective and affordable option for treatment or risk management [28, 29].

Second, our directive coaching script may have interfered with pharmacotherapy and alternative medicine treatments. The script was intended not only to complement but also directly support conventional pharmacotherapy, and it is not clear why it might not have. Quitlines may need to periodically evaluate the effectiveness of scripts, and consider revisions when unexpected effects are observed.

### Mixed coaching strategies

In practice, mixed coaching strategies may be preferable to purely nondirective or directive strategies. For instance, most participants needed some objective information about pharmacotherapy. Our nondirective coaching mode included objective information and corrections of any significant pharmacotherapy mistakes that were discovered, arguably making it a mixed coaching strategy for this topic.

Nondirective coaching may be useful when a smoker has questions about unanticipated smoking cessation topics, such as trying hypnosis. Also, nondirective coaching allowed some participants to discuss pressing off-topic problems. Whether this benefited participants is unknown, but it did not significantly prolong contact time. A quitline could employ a small number of nondirective coaches to handle pressing off-script questions and novel smoking cessation topics, in collaboration with a large number of directive coaches.

### Limitations

Both organizations were health related. Strong anti-smoking sentiment may be more motivating in these settings than in others.

We could hire only a few coaches. We minimized risks of biases related to coach age, race, or cultural background by training coaches in both directive and nondirective coaching modes. Participants’ social support inventories indicated equal directive and nondirective support in both coaching modes, raising questions about coaches’ ability to switch between modes. Although previously successful in differentiating nondirective and directive interventions [13], the measure may have been insensitive in this context. Indeed, detailed analyses of call duration, topic coverage, and types of statements, as well as differences in cessation rates in some subgroups, indicate that the interventions were objectively different.

Results were not confirmed by cotinine testing due to a lack of response. While self-reported 7-day quit rates are common quitline outcome measures [30], lack of biochemical confirmation leaves uncertainty. Cotinine testing was the unique study event requiring a physical exchange. Participants may have felt that saliva collection was discordant with the relatively anonymous nature of the coaching intervention.

## Conclusion

Telephone based coaching for smoking cessation within the context of several workplace-based incentives for quitting was effective, with 28 % overall reporting abstinence at 6 to 12-month follow-up and with no reductions in benefit for low income or ethnic minority employees. Further, both conditions were equally effective in engaging low income and African-American employees. Both were also effective in helping African Americans to quit. Within this broadly successful program, the use of a nondirective coaching style amongst employees with above-median income was most effective. Future research should examine mixed coaching strategies designed to address level of readiness to quit, social distress, or need for didactic instruction, as with pharmacologic smoking cessation aides.

## Abbreviations

CART, classification and regression tree; CATI, computer assisted telephone interview; CSSI, coach social support inventory; FTND, fagerström test for nicotine dependence; PHQ, Patient Health Questionnaire; PPOS, patient practitioner orientation scale; QSU, questionnaire of smoking urges; RSEQ, relapse situation efficacy questionnaire.

## Footnotes

1 **Supervisor Survey** Smoking prevalence among school employees was unknown, but less than 10 % of employees admitted smoking on health surveys. We conducted a supervisor survey at the school from January to March 2011 to independently estimate smoking prevalence. Top-level department administrators received a survey. Survey recipients who directly or indirectly supervised more than 30 individuals were to report only on directly supervised employees and forward a blank survey to the supervisors amongst them. The survey thus cascaded through tiers of supervisors. At each tier, survey recipients anonymously reported the top-level department name, how many staff they supervised, how many primary appointment faculty they served, and how many smokers were in each group. Survey responses covered 52 % of employees. Only 369 (7.3 %) of 5,030 staff and 21 (1.4 %) of 1534 faculty members were identified as smokers, in agreement with other smoking prevalence estimates. These data implied that about 530 medical school employees smoked. The very low prevalence of smoking at the school explains most of the difference in enrollment between the school and the hospital.

2 **Training Procedures** Training of phone coaches entailed each of the following elements:

- Didactic presentations by MW, EF, GH, WS, and worksite employee health representatives.
- Readings regarding the substantive areas addressed during in-service training, including *7 Steps to a Smoke-Free Life* (E. Fisher, Wiley, 1998) and *How to Quit Smoking without Gaining Weight* (B. Marcus, J. Hampl, & E. Fisher, Pocket Books, 2004), and *Motivational Interviewing: Helping People Change* (W. Miller, S. Rollnick, 2012).
- Meetings with experienced peer coaches and other intervention workers who were involved in other research at Washington University (R21 CA 10172-01; R01 HL 72919).
- Role play of telephone counseling interactions
- Closely supervised pilot implementation of the protocols with initial participants

**Training Content Areas** Training of the phone coaches included content in the following areas:

- Two protocols for directive and nondirective interventions, rationales for their comparison, and key distinction between them
- Fundamental skills in counseling and provision of support
- Assessment of readiness to quit smoking, incorporating assessment of readiness into counseling, and tactics for promoting key behaviors appropriate to the patient’s readiness to change
- Characteristics of smoking and key factors in smoking cessation
- Strategies for addressing barriers to smoking cessation and to maintenance of abstinence after cessation

**Ongoing Supervision** Supervision of the telephone coaches included the following:

- Weekly meetings with GH to address issues that arise as part of the intervention
- As needed supervisory meetings with GH and MW or WS to assess problems and progress in meeting coaches’ training needs
- Periodic review of the elements of training listed above
- In service didactic presentations by key personnel to address intervention issues that arose during the study

Protocols for training and supervision of coaches were reviewed by a medical advisor, worksite employee health representatives, and MW and EF. **Computer-Based Intervention Prompting, Monitoring & Documentation** Coaches were taught to use software tools for identifying and tracking calls and documenting call content. Laptop computer software prompted and monitored all coaching contacts and attempted contacts with participants. Records included the nature of each contact (protocol based call, patient-initiated unscheduled call, left message, attempted but unsuccessful call), duration of call, assessed stage, topics covered, and plans for next contact. Contact records were synchronized regularly to support identification of overdue contacts, quality control and supervision of the coaches, and daily updates of newly enrolled participants. During calls, the laptop software also prompted coaches with specific suggested questions to ask and “talking points” for each topic and behavior. **Control of Intervention Differences** In designing the study, we considered varied approaches to fidelity to the planned interventions and to controlling differences between them. Assigning one or several phone coaches to each condition risked confounding differences between interventions with personality or skill differences between the coaches. We therefore explained to coaches in detail the reasons for the study and the potential pros and cons of directive and nondirective coaching so that they would implement each coaching mode with full commitment to its value for participants, recorded all calls, reviewed many calls, and transcribed and analyzed a subset of calls.

3 **Imputing missing income data** Missing income data in 29 records were singly imputed using two classification and regression trees. The more accurate tree used marital status, education, age at baseline, sex, race, body mass index, and job rank as classifiers. Splits were made until the tree reached an R^2^ of 0.8, then leaves with 3 or fewer observations were pruned. The second tree was similar except that it omitted body mass index and job rank, which also were missing in 9 records, and splits were stopped at an R^2^ of 0.6. Missing income values were imputed as the most probable value in the first CART, or the second CART if the first required missing data. In records with complete data, this algorithm classified income correctly in 81 % of cases, with an R^2^ of 0.39.
